# Developing a Volunteer Education Initiative for Older Adults’ Health

**DOI:** 10.1093/geroni/igaa057.2400

**Published:** 2020-12-16

**Authors:** Liz Seidel, Tara Cortes, Cinnamon St John

**Affiliations:** 1 NYU Rory Meyers College of Nursing, New York, New York, United States; 2 New York University, New York, New York, United States; 3 Hartford Institute for Geriatric Nursing, New York, New York, United States

## Abstract

Older adults need sufficient information to make healthy decisions and be active participants in their healthcare. Yet there is often a lack of information available. The Bronx Health Corps (BHC) was created to meet this need by providing older adults with usable knowledge on managing health conditions and promoting healthy behaviors in community-based settings. The BHC trained 175 volunteers, educated 2,065 older adults, with a total attendance of >5,000. Steps of creating a volunteer education initiative will be presented with qualitative and quantitative data utilization in implementation of the program. Focus groups with older adults noted challenges in addressing community health needs and the importance of reaching outside of healthcare settings to address the health of the community. Focus groups with Spanish speaking older adults and caregivers expanded knowledge on their attitudes toward the 4Ms and their ability to use that knowledge in interacting with their providers.

